# Tips for Harnessing the Educational Potential of Tumor Boards for Medical Students

**DOI:** 10.1007/s40670-024-02141-6

**Published:** 2024-08-13

**Authors:** Aaron Lawson McLean, Anna C. Lawson McLean, Stefanie Hartinger, Jakob Hammersen, Robert Drescher, Salome Schuldt, Christian Senft, Matthias Mäurer, Marcel A. Kamp, Irina Mäurer

**Affiliations:** 1 Department of Neurosurgery, Jena University Hospital, Friedrich Schiller University Jena, Am Klinikum 1, 07747 Jena, Germany; 2 Department of Neurology, Jena University Hospital, Friedrich Schiller University Jena, Jena, Germany; 3 Department of Oncology and Hematology, Jena University Hospital, Friedrich Schiller University Jena, Jena, Germany; 4 Department of Nuclear Medicine, Jena University Hospital, Friedrich Schiller University Jena, Jena, Germany; 5 Department of Radiation Therapy and Radiooncology, Jena University Hospital, Friedrich Schiller University Jena, Jena, Germany; 6 Centre for Palliative and Neuropalliative Care, Brandenburg Medical School Theodor Fontane, Campus Rüdersdorf, Rüdersdorf, Brandenburg, Germany; 7 Comprehensive Cancer Center Central Germany (CCCG), Jena/Leipzig, Germany

**Keywords:** Medical students, Tumor boards, Oncology, Interprofessional education, Active learning, Reflective practice, Case-based learning, Digital learning tools

## Abstract

This paper explores the underutilized educational potential of tumor boards as a platform for medical student education. Acknowledging the complexity and multidisciplinary nature of tumor boards, we propose 12 strategic interventions aimed at integrating undergraduate medical students into these meetings to enhance their learning experience. These strategies emphasize active student engagement, critical analysis, patient interaction, reflective practice, and the integration of digital learning tools, with a focus on fostering an in-depth understanding of team-based, patient-focused oncology care. The approach advocates for the inclusion of medical students in tumor board discussions, not merely as observers but as active participants, thereby providing them with a unique, real-world learning environment. By doing so, the paper argues for the significant benefits of such involvement, including improved understanding of evidence-based practice, patient-centered care, ethical considerations, and the dynamics of interprofessional collaboration. This integrated educational model aims to prepare future physicians with the competencies necessary for effective participation in interdisciplinary healthcare teams, highlighting the importance of experiential learning in the context of oncology and beyond. The strategies outlined in this paper offer a roadmap for medical educators seeking to enhance the educational value of tumor boards and contribute to the development of a collaborative, informed, and empathetic oncology workforce.

## Introduction

Tumor boards, characterized by their convergence of diverse medical professionals and complex patient cases, represent an untapped educational resource for undergraduate medical students. The multidisciplinary nature of these boards has been shown to improve patient outcomes across a spectrum of cancers, in both adult and pediatric settings, and in diverse socio-economic contexts, from high-income to resource-limited settings [1–6]. This global and inclusive perspective is particularly relevant for medical students, who will be practicing in an increasingly interconnected and diverse world [7].

Nevertheless, simply being present at these meetings is not enough to harness their educational potential. Navigating this complex learning environment requires a strategic, structured approach that goes beyond passive observation. It is essential to foster active learning, critical thinking, reflection, and a nuanced understanding of the roles and dynamics within a multidisciplinary team [8, 9].

This paper presents a roadmap of 12 strategies to achieve these educational goals. By engaging with the rich, real-world learning environment of tumor boards, students can gain a deep understanding of the collaborative, patient-centered, and evidence-based approach that underpins successful oncological care. These strategies serve as a guide to transform the tumor board experience into a dynamic, immersive learning journey for students, equipping them with the knowledge, skills, and attitudes necessary for effective contribution within a multidisciplinary team. The 12 strategies proposed in this paper are synthesized from a comprehensive review of existing literature and best practices.

## Tip 1

### Incentivize Active Engagement

Encourage students to participate actively by assigning tasks that align with their level of expertise — summarizing patient histories or presenting relevant research findings. Active engagement stimulates critical thinking, promotes problem-solving skills, and instills a sense of responsibility towards their learning journey [10]. Moreover, this participation fosters reflection on interprofessional interactions, encouraging students to consider how their contributions are influenced by the multidisciplinary team [11]. Research suggests that such active engagement in multidisciplinary meetings significantly enhances learning experiences [12].

## Tip 2

### Foster a Question-Friendly Environment Through Open Discussion Forums

Facilitate open discussion forums where students feel safe to ask questions, share insights, and express their thoughts about the tumor board meetings. These forums can be held in a round-table format, with a faculty member acting as a facilitator to ensure respectful and constructive dialogue. Such an environment encourages active participation, enhances understanding, and fuels critical thinking [13].

## Tip 3

### Deepen Learning Through Structured Case-Based Discussions

Enhance the learning experience with structured, post-meeting case-based discussions. Select complex, multi-faceted cases from tumor boards for in-depth exploration in smaller student-led discussion groups. These discussions can delve into the nuances of each case, allowing students to critically analyze the patient history, evaluate diagnostic options, devise potential treatment strategies, and anticipate challenges in patient management. They also provide a platform for students to reflect on how multidisciplinary inputs shape the understanding and management of a case, thereby highlighting the value of diverse professional perspectives in patient care [14].

## Tip 4

### Foster Skills for Patient Education and Counseling through Structured Clinical Experiences

Where possible, arrange for students to have direct interactions with patients whose cases are being discussed in the tumor board. These interactions could involve taking a patient history, conducting a physical examination, or discussing the patient’s experiences and perspectives about their illness. Such experiences can provide invaluable insights into the patient’s perspective, enhancing empathy and grounding students’ learning in real-world clinical practice [15, 16]. Supplement this learning with opportunities to interact with patients in a clinical setting under supervision, where students can see the real-life implications of the decisions made during tumor boards. These experiences will help students understand the critical role of patient-centered care in shaping clinical decisions and outcomes.

## Tip 5

### Implement Reflective Writing Exercises

Post-meeting reflective writing exercises can serve as a powerful tool for consolidating learning and fostering self-awareness [17]. Encourage students to maintain a reflective journal where they can record their observations, thoughts, and questions about each tumor board meeting. The journal entries can focus on a variety of aspects such as the complexity of the cases, the interprofessional dynamics observed, the ethical dilemmas encountered, and the decision-making processes followed. Additionally, they should reflect on their own contributions and interactions within the team and how these experiences are shaping their understanding of oncology and their professional development. The journals can be reviewed periodically with a faculty mentor to provide feedback and guide further learning. Reflective writing has been recognized in medical education literature as an effective method for promoting critical thinking, self-awareness, and professional growth [18, 19].

## Tip 6

### Integrate Digital Learning Tools for Augmented Learning Experiences

Incorporate digital learning tools specifically designed to augment the tumor board learning experience [20, 21]. Utilize online platforms for curating a library of key cases discussed in the tumor board, complete with anonymized patient histories, diagnostic images, pathology reports, and summaries of the decisions made [22]. This can serve as a valuable resource for self-directed learning and future reference. Further, create a protected online discussion forum dedicated to each tumor board meeting. Here, students can continue the discussion beyond the meeting, share additional research findings, pose unanswered questions, and engage in peer learning [8]. Faculty members can participate in these forums, providing guidance and feedback and addressing student queries. These digital initiatives can offer a flexible, diverse, and multifaceted learning environment, catering to the varied learning preferences of today’s digital-native students [23, 24]. By integrating digital tools into the tumor board learning experience, we can enhance student engagement, foster active learning, and ensure continuous feedback and improvement [25].

## Tip 7

### Showcase the Spectrum of Interprofessional Collaboration

Expose students to the various roles within the tumor board through shadowing experiences. These opportunities can demystify the unique contributions of each team member to patient care, enhance understanding of team functioning, and expose students to the nuances of team communication, hierarchy, and negotiation [26]. Reflection on these experiences can foster a deeper appreciation for the diversity of professional roles [27]. Such interprofessional education experiences have been highlighted in research as valuable in preparing future physicians for collaborative practice [28].

## Tip 8

### Demystify the Multidisciplinary Decision-Making Process

Transform the seemingly complex decision-making process of tumor boards into a structured learning experience [29]. Begin by deconstructing previous decisions made during tumor boards, spotlighting how patient preferences, clinical guidelines, research evidence, and professional expertise are collectively considered [8, 30]. Also, highlight the role of effective communication, mutual respect, and consensus building within the team [31]. Then, facilitate student participation in this process by creating mock decision-making scenarios, for example, through the implementation of tactical decision games, enabling them to apply these principles in a safe learning environment [32].

## Tip 9

### Cultivate Critical Reflection on Interprofessional Interactions through Reflective Debriefing

Promote a culture of critical reflection on the nature and quality of interprofessional interactions within the tumor board [33–35]. Implement structured reflective debriefing sessions after each tumor board meeting, where students can share their observations about the dynamics of teamwork, the communication patterns they noticed, and the process of collaborative decision-making. These sessions can be facilitated by a faculty member who can provide insights, clarify misconceptions, and guide students in deriving learning from these reflections.

## Tip 10

### Reinforce the Importance of Evidence-Based Practice through Research Appraisal Exercises

To nurture a culture of evidence-based practice, incorporate research appraisal exercises into the learning plan. Assign students recent research articles related to the cases discussed in the tumor board and guide them in critically appraising the methodology, results, and applicability of the research findings. This not only enhances their research literacy but also highlights the application of research in clinical decision-making [36, 37].

## Tip 11

### Emphasize Patient-Centered Care

Patient-centered care is a crucial aspect of modern healthcare and a key element in the proceedings of a tumor board [38]. While patients themselves may not be physically present in these meetings, their experiences, preferences, and needs are central to the discussions [39, 40]. As such, it is essential that students understand how to place the patient at the center of their learning experience within the tumor board context [15, 41]. Encourage students to review each patient’s history, imaging, pathology, and the psychosocial aspects of their case critically. Highlight how the unique aspects of each patient’s case — their comorbidities, lifestyle, personal preferences, and social circumstances — influence the diagnostic and treatment decisions made during the tumor board.

## Tip 12

### Highlight Ethical Considerations

In oncology education, particularly within tumor board discussions, it is crucial to emphasize holistic ethical considerations, for example, by integrating the concept of “total pain” alongside traditional ethical dilemmas. Total pain, a term coined by Cicely Saunders, refers to the multifaceted nature of pain experienced by patients, encompassing physical, psychological, social, and spiritual dimensions [42]. Use the cases to bring ethical considerations in oncological care such as informed consent, patient confidentiality, end-of-life decisions, and balancing treatment benefits and harms to the forefront [43]. Encourage students to reflect on these aspects in dedicated ethics sessions, guiding them to understand the complexity of patient suffering and ethical decision-making in oncology. This approach, incorporating both conventional ethical dilemmas and the broader concept of total pain, aims to develop healthcare professionals who are not only ethically astute, able to reflect on these issues, debate differing viewpoints, and develop their own reasoned stance, but also deeply empathetic towards the multifaceted suffering of their patients [44].

## Conclusion

In conclusion, the integration of medical students into tumor board discussions presents a multifaceted opportunity to enhance their learning experience, equipping them with the skills and knowledge necessary for effective interdisciplinary collaboration in oncology care. This paper has outlined a comprehensive strategy, centered around active engagement, structured learning, patient interaction, reflective practice, and the use of digital tools, to maximize the educational potential of tumor boards. These strategies aim to foster a deep understanding of the complexities of cancer care, emphasizing the importance of evidence-based practice, patient-centered approaches, and ethical considerations. By engaging students in this dynamic, real-world context, we not only prepare them for their future roles in healthcare teams but also contribute to the cultivation of a more collaborative, informed, and compassionate oncology workforce.

Nevertheless, it is essential to acknowledge the practical realities of tumor boards. These meetings are primarily work-focused, designed to discuss as many patients as efficiently as possible [45, 46]. This fast-paced environment can pose significant challenges for educational integration, such as time constraints and the need to balance thorough discussion with efficiency [47]. Recognizing these challenges, it is crucial for educators to strategically plan student involvement, ensuring that their educational needs are met without disrupting the primary objectives of the tumor boards. Solutions may include preparatory sessions, selective participation in discussions, and post-meeting debriefings to maximize learning while maintaining the efficacy of the meetings.

The emphasis on interprofessional education within tumor boards underscores the necessity for future physicians to navigate and contribute to multidisciplinary teams effectively. The skills developed through such exposure—critical thinking, effective communication, ethical reasoning, and empathetic patient care—are fundamental to the practice of medicine and transcend the specific context of oncology. As medical education evolves, the incorporation of such experiential learning opportunities as tumor boards will be crucial in preparing students for the complexities of modern healthcare delivery.

Ultimately, this approach aligns with the broader objectives of medical education to produce healthcare professionals who are not only clinically competent but also capable of addressing the multifaceted challenges of patient care in a collaborative, ethical, and patient-centered manner. The strategies presented herein, therefore, not only enhance the educational value of tumor boards for medical students but also serve as a model for integrating experiential learning across the medical curriculum. As we continue to explore and refine these methodologies, it is imperative that we evaluate their impact on student learning outcomes and patient care, ensuring that our educational practices remain aligned with the evolving needs of our healthcare systems and the patients they serve.

## Data Availability

All datasets generated and analyzed during the current study are included in this published article. No additional data are available.
